# Fabrication and Characterization of Whey Protein—Citrate Mung Bean Starch—Capsaicin Microcapsules by Spray Drying with Improved Stability and Solubility

**DOI:** 10.3390/foods11071049

**Published:** 2022-04-06

**Authors:** Xiuyun Zhang, Bo Zhang, Xiangzhen Ge, Huishan Shen, Xiangxiang Sun, Qian Zhang, Yifan Lu, Zhuangzhuang Sun, Wenhao Li

**Affiliations:** College of Food Science and Engineering, Northwest A&F University, Xianyang 712100, China; zhangxiuyun123415@163.com (X.Z.); zhangbo383@163.com (B.Z.); 18393810565@163.com (X.G.); shen1685778117@163.com (H.S.); sx1141151470@163.com (X.S.); z-grace@nwafu.edu.cn (Q.Z.); luyifan0222@163.com (Y.L.); sunzhuangz@nwafu.edn.cn (Z.S.)

**Keywords:** capsaicin, microcapsules, physicochemical properties, storage stability

## Abstract

Capsaicin was microencapsulated in six different wall systems by spray drying whey protein and citrate mung bean starch at various ratios (10:0, 9:1, 7:3, 5:5, 3:7, 1:9, 0:10) to improve its stability and water solubility and reduce its pungency. The morphological, rheological, storage stability, and physicochemical properties of capsaicin emulsion and capsaicin microcapsules were characterized. As a result, the yield of six capsaicin microcapsules was 19.63–74.99%, the encapsulation efficiency was 26.59–94.18%, the solubility was 65.97–96.32%, the moisture content was lower than 3.63% in all systems, and particle size was broadly distributed in the range of 1–60 μm. Furthermore, microcapsules with high whey protein content in the encapsulation system had an excellent emulsifier effect and wetness, smooth particle surface, and higher lightness (L*). Moreover, the system formed by composite wall materials at a ratio of whey protein to citrate mung bean starch of 7:3 had the highest retention rate and the best stability. The overall results demonstrate that whey protein combined with citrate mung starch through spray drying could be a promising strategy to produce microcapsules of poorly water-soluble compounds such as capsaicin.

## 1. Introduction

Chili peppers (*Capsicum annuum*), known for their hot and spicy flavor and belonging to the family Solanaceae, are among the most commonly consumed spices [1,2]. According to official statistics, the total planting area of Chinese pepper reached 21,474 km^2^ in 2019. As a result, pepper accounts for China’s largest vegetable planting area [3]. Capsaicin (trans-8-methyl-N-vanillyl-6-nonenamide), an oil-soluble pigment, is the principal component of chili peppers, accounting for around 50% of the total capsanthin. Its molecular formula is C_40_H_56_O_3_, with a molecular weight of 584.87 g/mol [4,5,6]. A benzene ring and a long hydrophobic carbon tail with a polar amide group constitute the chemical structure of capsanthin [7].

Capsaicin is commonly used in food, medical treatment, antimicrobials, etc., as it can promote digestion, benefit blood circulation, prevent chronic diseases, and treat diverse painful syndromes [8]. However, multiple unsaturated C=C bonds in the structure of natural capsaicin result in poor water solubility and poor stability in adverse conditions. Moreover, capsaicin can produce a feeling of burning in the mouth, respiratory system, digestive tract, and stomach after excessive use or long-term direct contact [9]. The intense spice limits the broad application of capsaicin. Thus, it is essential to reduce the irritability of capsaicin and improve its stability and water solubility.

Microcapsule technology is an attractive way to protect the core material from external influence, mask unpleasant flavors, and stabilize labile compounds [10]. Although there are various wall materials, it is vital to meet safety, edibility, and sufficient emulsification requirements and to isolate product components when applying them in the food or pharmaceutical industry. Common wall materials include carbohydrates, plant water-soluble gum, and proteins [11]. However, the ideal performance of the product cannot be achieved by a single wall material. Therefore, these materials are often used in combination to satisfy the demands of various products in practical applications.

Starch is one of the most widely used biopolymers. Starch-based microcapsules hold great promise for carrying active ingredients (such as preservatives, colorants, flavors, and nutrients) and provide good protection against oxidation. However, native starch in microcapsules lacks emulsification properties and weak flavor retention ability [12,13]. Therefore, modification is imperative for starch. Ju et al. [12] prepared microcapsules of essential oil with modified starch, and the results confirmed that it could prolong the shelf life of foods. Citrate starch is the esterification product of anhydride formed by starch dehydration with citric acid. It can improve the emulsification and film-forming properties of natural starch. Jeon et al. [14] successfully developed microcapsules by entrapping a plant extract in native starch and modified starch matrices through spray drying.

Whey protein plays a vital role in anti-oxidation; it also has good emulsification and film-forming properties [15]. In addition, whey protein has been reported to exhibit good encapsulation properties. Therefore, it has been used as a wall material in vitamin E, avocado oil, and lutein. In addition, it protects the lutein more effectively from color degradation during storage [16,17,18,19,20]. Thanks to its apparent advantages, the spray-drying method has been the most commonly used microencapsulation technology in the food industry for decades.

Because of the limitation of modified starch as a single wall material, it can be combined with protein. For example, Tontul and Topuz reported that the combination of modified starch and whey protein endowed flaxseed oil microcapsules with a high entrapment rate [21]. In addition, there are few reports on the preparation of capsaicin microcapsules with citrate starch in combination with whey protein at present, and the application is uncommon. Furthermore, microcapsules prepared by composite wall materials can make up for the deficiencies of a single wall material and improve the core material’s encapsulation efficiency (EE) and oxidation stability.

In the present work, whey protein compounded with citrate mung bean starch was used as complex wall materials to construct capsaicin microcapsules through spray drying. The impacts of the two wall materials on the microcapsule system’s morphology, physical properties, and stability were assessed, and the better wall material was chosen. Finally, principal component analysis (PCA) was used to compare component differences in the microcapsules. This work aimed to produce capsaicin microcapsules with reduced pungency and improved functional performance to expand the application of capsaicin.

## 2. Materials and Methods

### 2.1. Materials

Mung bean (Ganzhou Kangrui Agricultural Products Co., Ltd. Jiangxi, China), whey protein (purity > 80%; Hefei Bomei Biotechnology Co., Ltd. Anhui, China), medium-chain triglyceride (MCT; Shanghai Yuanye Biotechnology Co., Ltd. Shanghai, China), capsaicin (purity > 80%; Beijing Kairuiji Biotechnology Co., Ltd. Beijing, China), Nile red (purity > 98%; Shanghai Yuanye Biotechnology Co., Ltd.), DMSO, citric acid, absolute ethanol, phenolphthalein, sodium hydroxide, hydrochloric acid, and so on were chemically pure.

### 2.2. Extraction of Mung Bean Starch

Mung bean seeds were washed, put in warm water at 30–40 °C, and soaked at room temperature for 6–10 h. The soaked seeds were ground and sieved (100-mesh), and the material under the sieve was collected and stirred thoroughly. Then, the starch slurry was allowed to settle for 4–5 h, after which the upper layer of water was removed, and water was added to the settled starch. After thoroughly stirring, the solution was passed through a 200-mesh sieve to collect the material under the sieve. The under-sieve material was repeatedly washed and settled with water until the top slurry layer was transparent. The supernatant was removed, and the sunken starch was collected. The obtained starch was dried at 40 °C, ground with a high-speed universal pulverizer, and sieved (100-mesh) to obtain a sample of mung bean starch [22].

### 2.3. Preparation of Citrate Starch

Citrate starch was obtained based on Lee et al. [23] with little modification. In summary, 30.0 g of citric acid (30% of the dry weight of starch) was dissolved in 60 mL of deionized water, and the pH of the solution was adjusted to 3.5 with 10 mol/L NaOH and diluted to a final volume of 120 mL. The above solution was blended with 100.0 g of starch in a beaker, and the starch slurry was kept at 28 °C for 14 h. The mixture was dried at 60 °C for 8 h to 5–10% (*w*/*w*). Next, it was transferred into a stainless-steel tray and dried at 130 °C for 5 h, and the dry mixture was repeatedly washed with deionized water. Finally, the washed starch was dried, ground, and sieved (100-mesh) again, then stored in a vacuum bag. The degree of substitution (DS) of mung bean starch modified by citric acid was 0.167 ± 0.004.

### 2.4. Preparation of Whey Protein–Citrate Mung Bean Starch–Capsanthin Emulsion

Protein was dissolved in deionized water and agitated for 12 h to allow it to completely swell. Modified starch was dissolved in deionized water, heated for half an hour at 70 °C with continuous stirring, and then stirred for 12 h at 25 °C. The modified starch was mixed with protein and stirred for 40 min to obtain an aqueous phase with 396 g. The ratio of protein mass to esterified starch was 10:0, 9:1, 7:3, 5:5, 3:7, 1:9, and 0:10. The protein was added with modified starch as a composite emulsifier, and its mass concentration was 5%; the mass concentration of the oil phase was 1%.

According to the preliminary experiment, capsaicin (100 mg, 0.5% of the wall material mass) was added to MCT (4.0 g) and heated at a high temperature (180 °C) until it dissolved to obtain the oil phase. After cooling, the oil phase was added slowly to the aqueous phase and stirred for 10 min. Finally, the above oil–water mixture was homogenized (6 min, 10,000 r/min) and ultrasonically treated (360 W, ≤45 °C) for 5 min to obtain the capsaicin emulsion.

### 2.5. Preparation of Whey Protein–Citrate Mung Bean Starch–Capsaicin Microcapsules

Capsanthin microcapsule powder was obtained after spray drying (inlet and outlet temperatures were 185 ± 5 °C and 85 ± 5 °C, respectively; feeding rate was 400 mL/h) [24]. The ratio of whey protein to citrate mung bean starch in the sample was 0:10. The oil phase and water phase were separated after spray drying and failed to form microcapsules. The preparation process was carried out under dark conditions with the sample wrapped in tin foil.

### 2.6. Morphology and Rheological Properties of Capsaicin Emulsion

#### 2.6.1. Morphological Observation of Capsaicin Emulsion

The morphology of the capsaicin emulsion was observed with an optical microscope (DMBA400, Macaudy corp., Xiamen, China) at a magnification of 400×.

#### 2.6.2. Fluorescence Microscope Observation of Capsaicin Emulsion

First, the fluorescent dye solution was obtained by dispersing 1 mg of Nile red (excitation wavelength at 488 nm) in 1 mL of DMSO. Next, 20 μL of dye solution was mixed with 0.5 mL of freshly prepared emulsion, and then 9 μL was placed on a glass slide, covered with a cover glass, and allowed to stand for 2 h. After natural drying, the distribution of oil phase was observed under the 20× objective lens of the fluorescence microscope (LECIA DM6 B, Lecia corp., Wetzlar, Germany).

#### 2.6.3. Rheological Properties

The rheological properties of capsaicin emulsion were measured by adding 1.25 mL of capsaicin emulsion to a DHR-1 rotary rheometer (Waters corp., Milford, MA, USA). The test conditions were as follows: the selection mode was flow scanning, the clamp was a 40 mm parallel aluminum plate, the temperature was 25 °C and 50 °C, and the shear rate was 0.01–100 s^−1^.

The Herschel–Bulkley model (τ = τ_o_ + kγ^n^) was applied to fit shear stress/shear rate curves, and the rheological parameters of the samples were obtained, where τ represents the shear stress, Pa; τ_o_ represents the yield stress, Pa; K represents the consistency coefficient, Pa·s^n^; γ represents the shear rate, s^−1^; n represents the fluid index; R^2^ represents the degree of fit [25].

### 2.7. Structural Characterization of Capsaicin Microcapsules

#### 2.7.1. Morphological Observation of Capsaicin Microcapsules

The morphology of the microcapsule particles was observed using a Nano SEM-450 scanning electron microscope (SEM) (FEI corp., Hillsboro, OR, USA). The dried sample was fixed on a carrier with conductive adhesive and sprayed with gold, observed, and photographed.

#### 2.7.2. Analysis of the Short-Range Ordered Structure of Capsaicin Microcapsules

FTIR spectra of the capsaicin microcapsules were scanned on a Vetex 70 Fourier transform infrared spectrometer (Bruker Corp., Karlsruhe, Germany) in the range of 4000 to 400 cm^−1^ at a resolution of 4 cm^−1^. Samples were ground and laminated with KBr at a ratio of 1:100. The test was carried out with a KBr sheet as the background.

### 2.8. Physicochemical Properties of Capsaicin Microcapsules

#### 2.8.1. Determination of the Yield of Capsaicin Microcapsules after Spray Drying

The yield of capsaicin microcapsules was calculated according to the following formula. The formula of yield (*Y*) is [26]:

(1) $$Y\;(\%)=\frac{M_{a}}{M_{b}}\;\times \;100$$

In the formula, *M_a_*: total mass of capsaicin in microcapsules, g; *M_b_*: total sample mass (capsaicin) added before spray drying, g.

#### 2.8.2. Determination of EE of Capsaicin Microcapsules

##### Drawing of the Standard Curve of Capsaicin

An accurately weighed 50 mg sample of capsaicin was dissolved in absolute ethanol and diluted to a final volume of 500 mL in a brown volumetric flask to obtain a standard capsaicin solution with a 10 mg/100 mL concentration. Gradient concentrations of 0.0, 1.0, 2.0, 3.0, 4.0, 5.0, 6.0, 7.0, 8.0, and 9.0 mg/100 mL were respectively prepared by using anhydrous ethanol to dilute standard solutions of 0.0, 1.0, 2.0, 3.0, 4.0, 5.0, 6.0, 7.0, 8.0, and 9.0 mL to 10 mL. The absorbance value was measured at a wavelength of 460 nm. The standard curve was drawn with the absorbance value as the ordinate and the concentration of capsaicin as the abscissa. The standard curve of capsaicin was plotted as follows: A = 0.0811C + 0.004 (R^2^ = 0.9999).

##### Determination of Capsaicin Content

The capsaicin content was determined by ultraviolet spectrophotometry. Determination of total capsaicin content in microcapsules: 100 mg of capsaicin microcapsule was dissolved in 5 mL of deionized water, placed in an ultrasound bath, and then put into a 100 mL brown volumetric flask with absolute ethanol at constant volume. The mixture was centrifuged (4 °C, 4000 r/min) for 10 min [12,26]. The absorbance value of the obtained clear solution was determined with a ZF-6 spectrophotometer (Jiapeng Technology Co., Ltd., Shanghai, China) at a wavelength of 460 nm, with aqueous ethanol solution (95%, *v*/*v*) as the control to calculate the total content of capsaicin.

Surface capsaicin was quantified by dissolving 200 mg of capsaicin microcapsules with 20 mL of absolute ethanol in a 50 mL centrifuge tube. After centrifugation (4 °C, 4000 r/min) for 10 min, 5 mL of supernatant was taken and diluted to 25 mL in a brown volumetric flask with absolute ethanol. The absorbance values were determined at a wavelength of 460 nm, with ethanol solution as the control to calculate the total content on the surface.

##### Calculation of EE of Microcapsules

The method of Kuang et al. [23] with a small adjustment was used to calculate the load rate:

(2) $$\mathrm{EE}\;(\%)=1-\frac{\mathrm{Surface}\;\mathrm{content}\;\mathrm{of}\;\mathrm{capsaicin}}{\mathrm{Total}\;\mathrm{capsaicin}\;\mathrm{content}}\;\times \;100$$

#### 2.8.3. Determination of Moisture Content of Capsaicin Microcapsules

The moisture content in microcapsules was determined by the vacuum-oven method.

#### 2.8.4. Solubility of Capsaicin Microcapsules

A certain mass of capsaicin microcapsules (dry basis) was dissolved in deionized water and centrifuged (4 °C, 4000 r/min) for 10 min, and the supernatant was poured out. The residue was transferred into an aluminum box and dried to constant weight. Solubility (*S*) was calculated using the following Equations:

(3) $$S\;(\%)=[1-\frac{M_{2}{-M}_{1}}{M}]\;\times \;100$$

where *M* is sample mass (g); *M_1_* is aluminum box mass (g); *M_2_* is the total mass of the insoluble substance and aluminum box (g).

#### 2.8.5. Wetness of Capsaicin Microcapsules

A 0.1 g sample of microcapsule powder was dispersed in 50 mL of deionized water and agitated continuously, and the time required for the powder to be entirely wetted was measured [27,28,29].

#### 2.8.6. Color Measurement

The chroma value of microcapsule particles was measured using a colorimeter (CI7600, Aisili Color technology Inc., Shanghai, China) calibrated with a black and white standard plate. A 10 mm reflection ring was selected as the measuring hole. The value of L* (lightness index scale) ranges from 0 (black) to 100 (white). The parameter a* represents the level of red (+a*) or green (−a*) color, and the value of b* measures the level of yellow (+b) or blue (−b*) color. All measurements were conducted three times for each sample. The 10W-0OP sample was taken as the standard sample to calculate the color difference (ΔE) value. The calculation formula is as follows:

(4) $$\Delta E=\sqrt{{\left(L^{*}-L_{0}^{*}\right)}^{2}+{\left(a^{*}-a_{0}^{*}\right)}^{2}+{\left(b^{*}-b_{0}^{*}\right)}^{2}}$$

In the formula, L*, a*, and b* represent the color of the capsaicin microcapsule sample; L_0_*, a_0_*, and b_0_* represent the color of microcapsule samples prepared with protein as a single wall material.

#### 2.8.7. Measurement of Particle Size Distribution

The particle size of capsaicin microcapsules was measured through laser particle size analyzer (LS13320, Beckman Coulter Corp., Brea, CA, USA), and the microcapsule samples were dissolved in deionized water. The universal liquid module (ULM) was selected as a sample processing module.

#### 2.8.8. Thermal Analysis

The thermal properties of the capsaicin microcapsules were analyzed by a differential scanning calorimeter (Q2000; Waters Corp., Milford, MA, USA) under a nitrogen atmosphere at a flow rate of 20 mL/min. A 3 mg sample was hermetically sealed in an aluminum pan and heated from 30 °C to 250 °C at a heating rate of 10 °C/min to obtain the DSC curve, and an empty hermetic pan was used as a control. The DSC curve was processed and analyzed with TA software.

### 2.9. Storage Stability of Capsaicin Microcapsules

#### 2.9.1. Effect of Temperature on the Retention Rate of Capsaicin Microcapsules

The three kinds of capsaicin microcapsules with the highest EEs were stored in a dark and sealed environment at 25 °C or 50 °C for 15 day, and a 100 mg sample was taken every 3 day to determine the total capsaicin content and calculate the retention rate (*R*) of capsaicin in the microcapsules. The following formula was used to calculate the retention rate:

(5) $$R\;(\%)=\frac{\mathrm{Residual}\;\mathrm{capsaicin}\;\mathrm{content}}{\mathrm{Initial}\;\mathrm{capsaicin}\;\mathrm{content}}\;\times \;100$$

#### 2.9.2. Effect of Light on the Retention Rate of Capsaicin Microcapsules

The three kinds of microcapsules with the highest EEs were sealed and stored under ultraviolet light or natural light for 15 day at room temperature, and a 100 mg sample was taken every 3 day to determine the total capsaicin content and calculate the *R* of capsaicin in the microcapsules.

### 2.10. Data Processing

All experiments were carried out in triplicate, and the final results are presented as the mean ± standard deviation. The data were processed with Excel 2013, Minitab 18.1 was used for statistical analysis (Tukey, *p* < 0.05), SPSS 20.0 was used for principal component analysis (PCA), and Origin 8.0 was used for graphing.

## 3. Results and Discussion

### 3.1. Morphological Structure of Capsaicin Emulsion

**Figure 1:**
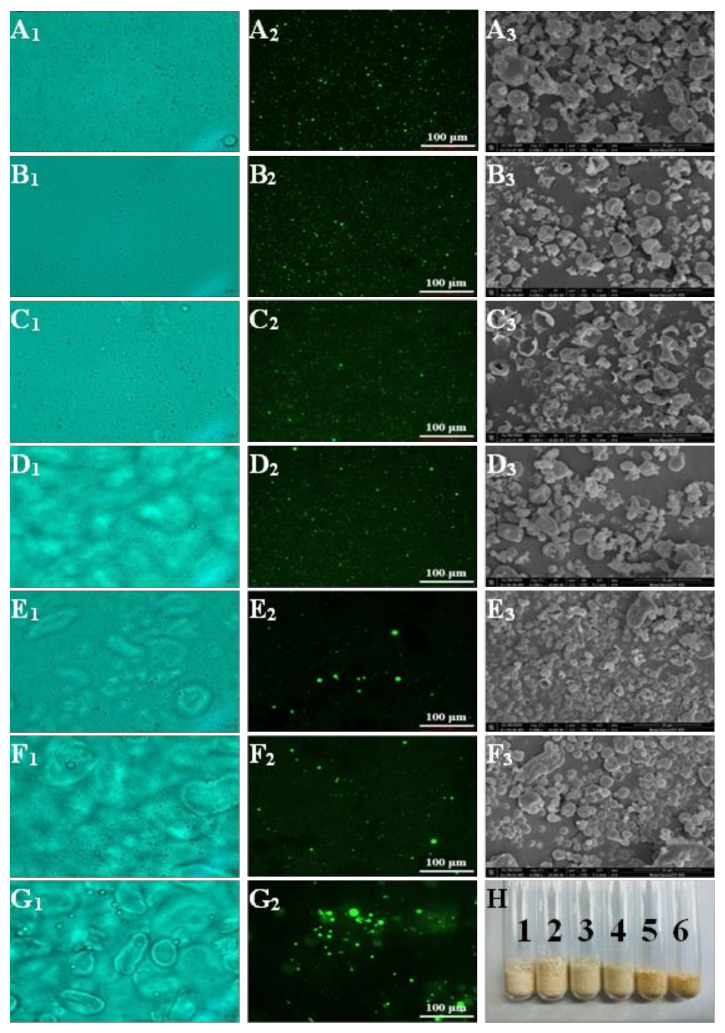
Optical micrographs (×400) (1) and fluorescence micrographs (FM) (×200) (2) of capsaicin emulsion. Scanning electron micrographs (SEM) (×6000) (3) and appearance (**H**) of microcapsules. (**A**–**G**) represent whey protein to citrate mung bean starch ratios of 10:0, 9:1, 7:3, 5:5, 3:7, 1:9, and 0:10, respectively. In the figure showing the appearance of microcapsules, samples 1 to 6 represent whey protein to citrate mung bean starch ratios of: 10:0, 9:1, 7:3, 5:5, 3:7, and 1:9, respectively.

Figure 1A_1_–G_1_ show the morphology of the capsaicin emulsion under an optical microscope. The droplets with high whey protein content were tiny and evenly distributed, which shows that the emulsion had an excellent emulsifying effect and can effectively prevent the fusion between droplets and form a more stable emulsion. With the increase in citrated mung bean starch content in the emulsion (ratios of whey protein to citrate mung bean starch were 5:5, 3:7, and 1:9), the tiny oil droplets aggregated together. Small oil droplets adhered to each other or grew on the surface of large oil droplets to be fused, which indicates that emulsions emulsified with more citrate mung bean starch were poor. Kierulf et al. [30] also mentioned that protein has better emulsifying properties than starch. Compared with other emulsions, the emulsion stabilized by only citrate mung bean starch had larger droplets and aggregates that could be observed with the naked eye. The results show that the emulsifying effect of citrate mung bean starch alone is poor and cannot stabilize the emulsion very well.

### 3.2. Fluorescence Microscope

The fluorescence microscope (FM) observes the distribution of the oil phase in the emulsion by staining the oil phase specifically. Capsaicin was dissolved in MCT and stained with Nile red (labeled green). The FM photographs of whey protein–citrate mung bean starch–capsaicin emulsion are shown in Figure 1A_2_–G_2_. This figure shows that oil droplets in the emulsion system with more protein content were smaller and evenly distributed, which shows that the emulsifier has a better emulsifying effect. This further demonstrates that protein is an excellent emulsifier [31]. When the ratio of whey protein to citrate mung bean starch was 1:9 and 3:7, large aggregates and droplets appeared in the emulsion. The distribution of droplets was very uneven, indicating that the emulsifier emulsified with more citrated mung bean starch content has a poor emulsification effect. The tiny droplets in the emulsion stabilized by citrate mung bean starch alone assembled into clusters. The droplets in the emulsion became more visible, demonstrating that some of the droplets had aggregated and fused. The results indicate that the use of only citrate mung bean starch leads to poor emulsifying properties.

### 3.3. Rheological Properties

**Figure 2:**
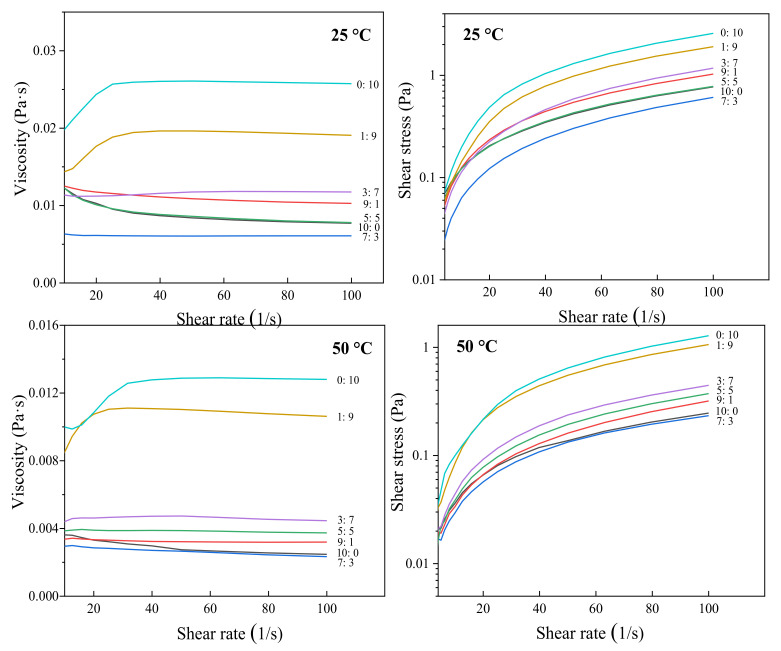
Shear rheological curve of capsaicin emulsion. The labels 10:0, 9:1, 7:3, 5:5, 7, 1:9, and 0:10 represent the ratio of whey protein to citrate mung bean starch ester.

The shear rheological behavior of the capsaicin emulsion at a shear rate of 0–100 s^−1^ at 50 °C and 25 °C is displayed in Figure 2. The figure shows that the apparent viscosity of the emulsion system increased rapidly at a low shear rate when the ratio of whey protein to citrate mung bean starch was 0:10 and 1:9. In contrast, the apparent viscosity of the other systems decreased as the shear rate increased and showed shear-thinning behavior, which is a representative characteristic of non-Newtonian fluids [32]. Figure 2 shows the function relation between shear stress and the shear rate at different temperatures. The emulsion system had various degrees of convexity on the shear stress axis, which indicates that the sample emulsion behaved as a pseudoplastic fluid. Zhang et al. [33] mentioned that many factors could affect the viscosity of protein solutions, such as the solvent, surface charge, shear rate, temperature, etc. At the same temperature, except when the ratio of whey protein to citrate mung bean starch was 9:1 and 7:3, the apparent viscosity and shear stress of the system decreased with increasing whey protein content in the compound emulsifier. The system with higher viscosity has stronger intermolecular forces.

The Herschel–Bulkley equation (τ = τ_o_ + Kγ^n^) was used to fit the obtained shear rheologic curve. The fitting results are recorded in Table 1; the values of the coefficient of determination (R^2^) ranged from 0.9960 to 0.9997, which highlights that the model is very suitable for fitting the rheological curves of this study [34,35,36]. In this study, when the ratio of whey protein to citrate starch was 10:0, 9:1, 7:3, and 5:5 at 25 °C and the ratio of whey protein to citrate mung bean starch was 10:0 and 7:3 at 50 °C, it behaved as a pseudoplastic fluid for all flow behavior indices *n* < 1.

When the ratio of whey protein to citrate mung bean starch was 1:9 and 0:10, the consistency coefficient (K) was significantly higher than those of other emulsion systems (*p* < 0.05), indicating that the system with more modified starch content could form a strong and compact three-dimensional network structure, which improved the thickening effect. At the same time, it also showed the interaction between starch and protein, which improved their compatibility [37]. Furthermore, the consistency coefficient (K) of capsaicin emulsion at 25 °C was higher than that at 50 °C. The fluid index (n) at 25 °C was lower than at 50 °C, suggesting that temperature can affect the viscosity and fluidity of the emulsion. Chin et al. [38] found that temperature and concentration could significantly affect the index and consistency coefficient behavior, and viscosity increased with temperature and increased with total soluble solid content. Strong linkages between protein and modified starch can form at low temperatures to stabilize the emulsion system. Therefore, the tensile deformation degree of the emulsion system was insufficient under the action of stress, resulting in the emulsion system having a lower n value and a higher K.

### 3.4. Morphological Structure of Capsaicin Microcapsules

The appearance and morphology observed with SEM of the six microcapsule systems composed of whey protein and citrate mung bean starch are shown in Figure 1. The dispersity and color of capsaicin microcapsules were homogeneous, and the powder was fluffy and fine. There was no accumulation, highlighting that the quality of the powder formed by spray drying is good. Most of the powders were well separated and did not stick together; the shape of each microcapsule particle was almost spherical, and the surface membrane structure on the sphere was compact and consecutive. Li et al. [39] pointed out that small starch granules form spherical aggregates when spray-dried in the presence of proteins. However, the particle size was not remarkably homogeneous. In addition, wrinkles and dents were observed around some of the microcapsules (Figure 1A_3_–F_3_). This may be caused by the rapid dehydration of the emulsion during spray drying, which results in the shrinkage of the wall materials of the microcapsules, leading to dents around the powder.

When the ratio of whey protein to citrate mung bean starch was 10:0, 9:1, and 7:3, the surface of the microcapsules was smooth. They also exhibited a high degree of integrity with no noticeable wrinkles or cavities on the surface. In addition, the EE of these microcapsules was favorable, indicating that the capsaicin was well encapsulated. However, with the increased content of modified starch in the wall materials, some holes appeared on the surface of some microcapsules. Generally, holes can enhance the permeability of microcapsules and weaken the microencapsulation degree, thus leading to the oxidation and degradation of microcapsules, which become easily affected by the environment [26]. Furthermore, the moisture evaporation rate of citrate mung bean starch in the wall materials was fast. As a result, the wall materials expanded rapidly, which damaged the surface structure of the microcapsules, leading to cracks and holes on the surface. Moreover, microcapsule particles with a large proportion of modified starch in the wall materials had poor uniformity, and some particles appeared to be cohesive [40].

### 3.5. Short-Range Ordering of Capsaicin Microcapsules

**Figure 3:**
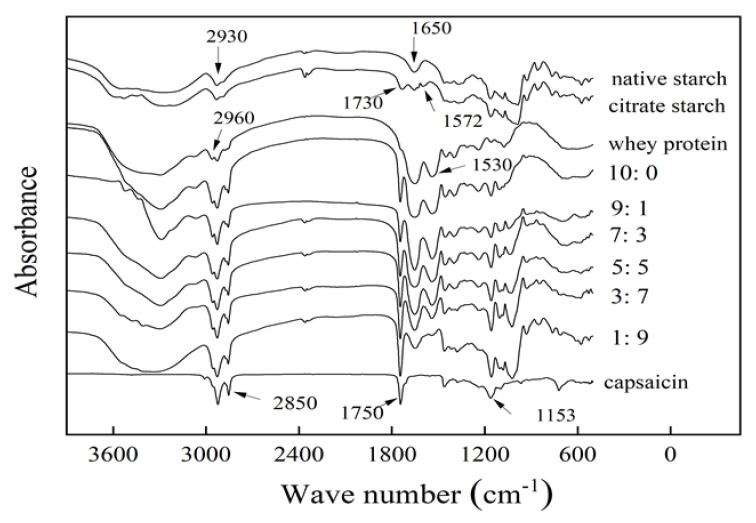
FTIR spectra of whey protein-citrate starch-capsaicin microcapsules. The labels 10:0, 9:1, 7:3, 5:5, 3:7, and 1:9 represent the ratio of whey protein to citrate mung bean starch.

The Fourier transform infrared spectrometer (FTIR) detects whether capsaicin microcapsules introduce new functional groups or form new chemical bonds, and the molecular structure can be characterized. The obtained infrared spectrum is shown in Figure 3. The infrared spectra of natural mung bean starch and citric acid-modified starch are similar. The broad peaks at 3200–3600 cm^−1^ of natural mung bean starch represent the characteristic vibrational stretches of –OH groups [41]. The stretching vibration of –CH was observed at 2930 cm^−1^; a strong absorption peak observed at 1650 cm^−1^ is ascribed to water combined with starch.

For citrate mung bean starch, the absorption peaks observed at 1730 cm^−1^ and 1572 cm^−1^ correspond to functional group vibrations of –C=O and RCOO–, respectively. This proves that the starch introduced new groups after esterification and verifies the esterification of native starch with citric acid in an alkaline environment. The sharp and tiny peaks at 2960 cm^−1^, 2930 cm^−1^, and 2850 cm^−1^ of whey protein represent –CH stretching and bending vibration bands; there were two prominent characteristic peaks at 1530 cm^−1^ and 1650 cm^−1^. In addition, the spectrum contained peaks at 1153 cm^−1^, 1750 cm^−1^, 2850 cm^−1^, and 2930 cm^−1^, which are characteristic absorption peaks of pure capsaicin.

**Figure 4:**
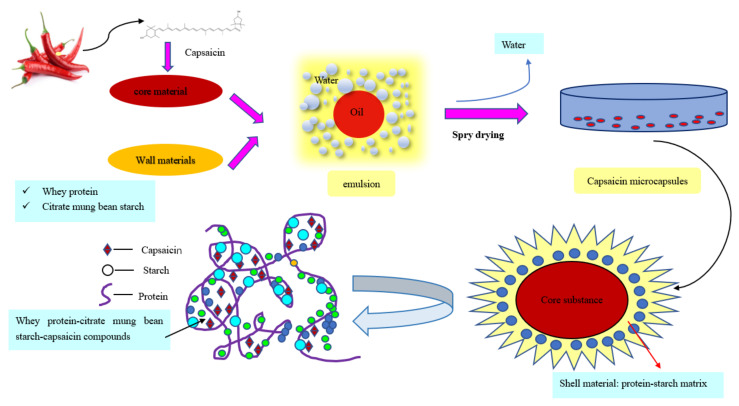
Mechanism of capsaicin encapsulation by starch and protein.

In addition to the characteristic peaks of the wall materials in the microcapsules, there were also more substantial absorption peaks near 1750 cm^−1^ than pure capsaicin, with greater intensity and narrower peaks caused by C=O stretching vibration. This is connected to the encapsulation of capsaicin; it also indicates that the active components of capsaicin formed strong bonds with the compound materials and could form a stable microcapsule system. The mechanism of capsaicin encapsulation by starch and protein is shown in Figure 4.

Moreover, the absorption peaks of microcapsule powder products at approximately 2850 cm^−1^, 2930 cm^−1^, and 2960 cm^−1^ were sharper and stronger than the characteristic peaks of whey protein. In addition, a distinct peak was formed at 1153 cm^−1^, which was related to capsaicin microcapsules.

### 3.6. Yield, EE, Moisture Content, Solubility, and Wetness of Capsaicin Microcapsules

The yield, EE, moisture content, solubility, and wetness of the capsaicin microcapsules after spray drying are recorded in Table 2. As shown in Table 2, the yield of the six microcapsules ranged from 19.63% to 74.99%. With the increase in the citrate mung bean starch content in wall materials, the yield of the microcapsule system decreased significantly (*p* < 0.05). When the ratio of whey protein to citrate mung bean starch was 1:9, the yield was the lowest, and the water content reached the peak (Table 2), which demonstrates that the microcapsules formed by the compound wall materials were not easily dehydrated, had high viscosity, and readily stuck to walls [42].

The EE of capsaicin microcapsules ranged from 26.59% to 94.18%. When the ratio of whey protein to citrate mung bean starch was 3:7 and 1:9, the EE was 58.65% and 26.59%, respectively, which are extremely low, indicating that the microcapsule system with a higher content of citrate mung bean starch in composite wall materials had poor emulsification performance and encapsulation effect. The EE of the other four kinds of powder was higher than 81.53% and reached a higher level. There is high capsaicin content on the surface of the microcapsules with lower EE, which is easily oxidized by the environment and will affect the integral quality of the microcapsule powders by reducing the solubility, wetness, stability, etc.

In addition, it can be seen in Table 2 that the moisture content of all six kinds of microcapsule products was lower than the maximum water content of dry powder in the food field of 3.00–4.00%, reaching a relatively low level. For example, the maximum moisture content of the samples was 3.63% when the ratio of whey protein to citrate mung bean starch was 1:9, and there was no significant difference in the moisture content among the other five microcapsules (*p* < 0.05). The shallow moisture content makes the microcapsules less susceptible to mildew, absorption of moisture, and aggregation; decreases the flow and dispersity of active constituents; reduces the possibility of microbial growth and lipid oxidation; and improves the storage stability of capsaicin. Higher moisture content may be ascribed to the rapid formation of the shell structure of the modified starch during the faster drying process. As a result, the internal water did not diffuse to the surface in time, making it hard for the moisture to evaporate.

Solubility is a crucial element in measuring the quality of powder ingredients. Products with poor solubility are not easily processed and will cause economic losses. As shown in Table 2, capsaicin microcapsules had a high solubility between 65.97% and 96.32%, and the highest solubility of samples produced with only whey protein as the wall material was 96.32%. With the increase in citrate mung bean starch in wall materials, the solubility of microcapsules markedly decreased (*p* < 0.05). The hydrophobic properties of citrate mung bean starch resulted in decreased solubility of the capsaicin microcapsule powders with the addition of citrate starch [43]. Natural capsaicin is insoluble in water and is a fat-soluble pigment that is difficult to use in the food industry. Therefore, the solubility of capsaicin microencapsulated with whey protein and citrate mung bean starch as wall materials is improved. The core material can be released after the product contacts water and can then play its role.

The wetness of microcapsules refers to the capacity of powder to be wetted by water to reflect its rehydration properties, usually recorded as the time that it takes for the powder to completely dissolve in water [44]. It was reported that the wetness is related to the particle size of the system; a small particle size results in a large specific surface area, making it easier to interact with water, thus increasing the wetness of the microcapsules and reducing the time required for the powder to dissolve in water [44]. Chew et al. [45] pointed out that the shorter the dissolution time of powder in food processing, the better the physical properties. Therefore, the wetness of microcapsules was better when the whey protein content in the encapsulation system was high. As a result, the time required for the microcapsule powders to completely dissolve in water was reduced (Table 2), which indicates that the whey protein required the shortest time to dissolve in water, and the citrate mung bean starch required a longer time.

### 3.7. Color of Capsaicin Microcapsule

The color values (L*, a*, b*) and ΔE of capsaicin microcapsule powder prepared with the combination of whey protein and citrate green bean starch are shown in Table 2. We can see that the capsaicin microcapsules were pale orange powders in appearance. When the ratio of whey protein to citrate mung bean starch was 10:0, 9:1, and 7:3, the lightness of microcapsules was significantly higher than the others (*p* < 0.05), which were 93.38, 93.49, and 92.59, respectively. Meanwhile, we observed that the particle sizes of the two samples with ratios of whey protein to citrate mung bean starch of 10:0 and 9:1 were also the smallest. Other researchers have found a similar phenomenon in studies of pigment powders and red pepper powders; the L* values increased as the particle size decreased [46]. This may be attributed to the fact that powders with smaller particles are more tightly packed, showing more surface in a given exposed area and therefore reflecting more light [46]. There was no significant difference in L* values among the three microcapsules. A significant decrease (*p* < 0.05) in the L* values was observed for the other samples as the whey protein content in the wall materials gradually decreased. The samples with higher whiteness indicate that the less the amount of capsaicin exposed on the surface of microcapsules, the better the EE, which is accordant with the above EE results (Table 2).

The values of a* and b* increased with the proportion of modified starch in the encapsulation system (except for ratios of whey protein to citrate mung bean starch of 10:0 and 9:1). There was a significant difference between the samples (*p* < 0.05). When the ratio of whey protein to citrate mung bean starch was 1:9, the a* value reached a maximum of 14.87, indicating that the color tended to be red; under such conditions, the b* value also reached the maximum of 34.86, illustrating that the color of the product tended to be yellow. In terms of ΔE, there was no apparent difference between samples with ratios of whey protein to modified starch of 10:0 and 9:1; when the ratio of whey protein to citrate mung bean starch was 7:3, 5:5, 3:7, and 1:9, there was a significant difference from the first two types of microcapsules (*p* < 0.05). The difference increased with decreasing whey protein content in the wall materials, showing that the color of the microcapsules tended to be dark orange.

### 3.8. Determination of Particle Size Distribution of Capsaicin Microcapsules

The mean particle size and particle size distribution of capsaicin microcapsule powder formed by spray drying are recorded in Table 2. Microcapsules with a high content of whey protein had a smaller particle size. The (d_4,3_) and (d_3,2_) of microcapsules with whey protein as the only wall material were 1.12 μm and 0.83 μm, respectively. When the ratio of whey protein to citrate mung bean starch was 9:1, (d_4,3_) and (d_3,2_) were 1.20 μm and 0.87 μm, respectively. With the increasing content of modified starch in the wall materials, the mean grain size of microcapsules markedly increased (*p* < 0.05). A high-viscosity emulsion can form larger droplets during atomization. The particle size of the microcapsule powder was increased after spray drying, corresponding to the viscosity observed in the analysis of the rheological properties of the capsaicin emulsion described above. This may be due to increased particle size distribution at higher drying air temperatures [47]. The microcapsule powder made by spray drying in this study is micron-sized. In the preparation process of the capsaicin emulsion, the high-speed shearing and ultrasonic waves will increase the interaction between modified starch and whey protein and break the interface resistance between the wall materials and the core material, promoting the bonding of the three ingredients in the system (Table 2).

In terms of the three microcapsule products with higher EE (ratio of whey protein to mung bean starch citrate was 10:0, 9:1, and 7:3), when the ratio of whey protein to citrate mung bean starch was 7:3, the mean particle size of the capsaicin microcapsule powders was more extensive, which was helpful in improving the stability of microcapsules. The results are consistent with the storage stability of capsaicin microcapsules described below. Smaller particles have a larger specific surface area, increasing the possibility of contact between microcapsules and the external environment and decreasing the stability of microcapsule products.

In addition, the viscosity of the emulsion also affects the particle size distribution of microcapsule particles. It was previously reported that as the viscosity of the solutions increased, the number of large particles increased, while the number of small particles decreased [48]. In general, the particle size of microcapsules is not homogeneous and is widely distributed. The capsaicin microcapsules were commonly distributed in the range of 1–60 μm. When the ratio of whey protein to citrate mung bean starch was 10:0 and 9:1, the d _(3,2)_ and d _(4,3)_ of microcapsules were close to each other, indicating that the particle shape of microcapsules was more regular compared to the others. The particle size was more uniform, while the particle size uniformity of other products was poor, and the distribution range was wide.

### 3.9. Thermal Property Analysis of Capsaicin Microcapsule

**Figure 5:**
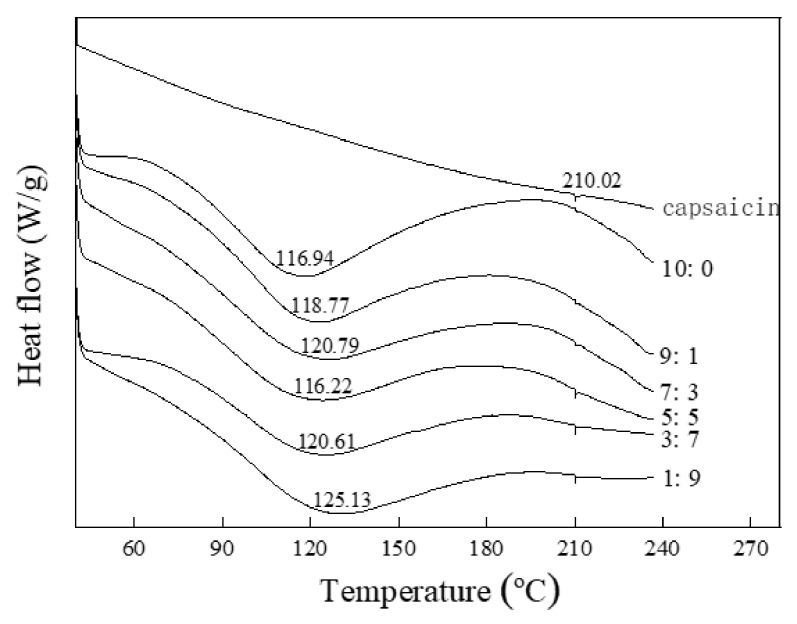
DSC thermograms of whey protein–citrate starch–capsaicin microcapsules. The labels 10:0, 9:1, 7:3, 5:5, 3:7, and 1:9 represent the ratio of whey protein to citrate mung bean starch.

Using TA software to analyze and compare the obtained DSC curves to study the thermal properties of capsaicin microcapsules is of great significance in evaluating product quality, safety, and stability. The DSC curves and glass transition temperatures of the six capsaicin microcapsules are presented in Figure 5.

The dissolution temperature of natural capsaicin was 210.02 °C. The six capsaicin microcapsules formed after spray drying showed the first endotherm peak near 120 °C, the glass transition temperature (T_g_). This demonstrates that the glass state of capsaicin microcapsules could remain stable at room temperature. Thus, the structure of capsaicin microcapsules could remain intact after heat treatment, which might be attributed to the compound having a stable structure composed of starch, protein, and capsaicin. Compared with the first four microcapsules with higher EE, when the ratio of whey protein to citrate mung bean starch was 7:3, the maximum glass transition temperature of the product was 120.79 °C. This indicates that the microcapsules required the highest amount of heat for phase change and also implies that the microcapsules prepared with composite wall materials have good thermal stability.

### 3.10. Storage Stability of Capsaicin Microcapsules

**Figure 6:**
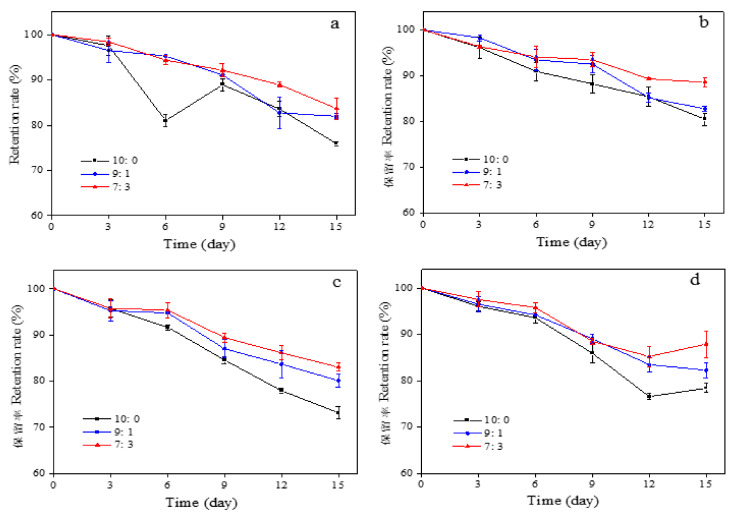
The effect of different storage conditions ((**a**) 50 °C in the dark; (**b**) 25 °C in the dark; (**c**) UV light at room temperature; (**d**) sunlight at room temperature) on the retention rate of microcapsules. The labels 10:0, 9:1, 7:3, 5:5, 3:7, and 1:9 represent the ratio of whey protein to citrate mung bean starch.

The storage stability results of whey protein–citrate mung bean starch–capsaicin microcapsules at different temperatures are shown in Figure 6a,b. The temperature significantly affected capsaicin microcapsules. After 15 day of storage at 50 °C, the retention rates of microcapsules were 75.91%, 81.96%, and 83.68% when the ratio of whey protein to citrate mung bean starch was 10:0, 9:1, and 7:3, respectively. After 15 day of storage in the dark at 25 °C, the retention rates of microcapsules were 80.51%, 82.80%, and 88.57% when the ratio of whey protein to mung bean starch citrate was 10:0, 9:1, and 7:3, respectively. The results indicate that the microcapsules prepared by spray-drying technology have specific heat resistance, and Xiao et al. [49] found that the retention rate of capsanthin decreased with the rise in temperature. The system formed by the composite wall materials with a ratio of whey protein to citrate mung bean starch of 7:3 was better at improving the heat resistance of capsaicin.

The retention rate of microcapsules under different light conditions is shown in Figure 6a–d. After 15 day at room temperature under UV light, the retention rates of three capsaicin microcapsules (whey protein to citrate mung bean starch ratios of 10:0, 9:1, and 7:3) were 73.17%, 80.11%, and 83.01%, respectively. Meanwhile, after 15 day of storage at room temperature under sunlight, the retention rates of the three microcapsules were 78.38%, 82.26%, and 87.89%, respectively. The retention rates of capsaicin microcapsules were all above 70.00% after storage for 15 day under different light conditions, which indicates that capsaicin microcapsules have a good photoprotective effect on capsaicin. In addition, when the ratio of whey protein to mung bean starch citrate was 7:3, it effectively improved the photostability of capsaicin.

### 3.11. Principal Component Analysis

**Figure 7:**
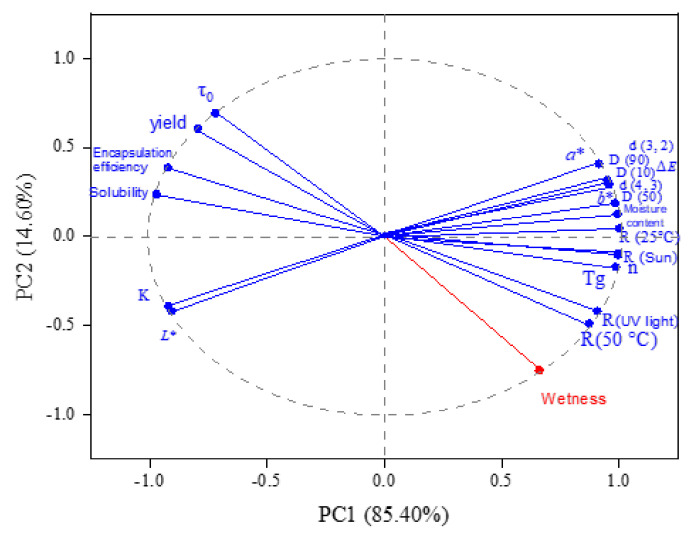
Principal component analysis of group differences.

Principal component analysis (PCA) compares the differences among the components of capsaicin microcapsules. There is no variable directly related to the principal component. The contribution value of the first principal component (PC1) was 85.40%, which principally affected the yield of microcapsules, moisture content, EE, solubility, color value, particle size distribution, glass transition temperature, rheological properties, and storage stability. The contribution value of the second principal component (PC2) was 14.60% (Figure 7), which was mainly related to wetness. These results show that besides wetness, physicochemical and storage stability influenced each other and determined the properties of microcapsules together.

## 4. Conclusions

This study used capsaicin as the core substance and whey protein composited with citrate mung bean starch as wall materials to prepare oil-in-water-type microcapsules based on spray drying. The aim of this study was to improve the stability and water solubility of active components of the core material. The results show that capsaicin emulsion is a typical non-Newtonian fluid. The emulsification performance of the microcapsule system with high whey protein content was excellent, and the surface of the microcapsule particles was smooth. The yield, EE, and lightness (L*) were also increased compared to microcapsules with low whey protein content. The moisture content was lower than 3.63%, and the solubility of microcapsules increased significantly and decreased with the increase in citrate mung bean starch content in the wall materials (*p* < 0.05), which was attributed to the hydrophobicity of starch. Light and temperature significantly affected the retention rate of capsaicin microcapsules. Moreover, the system formed by the composite wall materials with a ratio of whey protein to mung bean starch citrate of 7:3 had the highest retention rate and the best stability.

These findings show that capsaicin encapsulation by spray drying whey protein mixed with citrate mung bean starch as wall materials is efficient. Capsaicin encapsulated in microcapsules has satisfactory stability, good water solubility, and reduced irritability and can be added to other foods to improve its functional properties. Our findings expand the application of capsaicin and reveal a new way to apply microcapsules with starch-based wall materials for delivering functional ingredients. There is great potential for designing food-grade delivery systems to encapsulate natural lipophilic pigments or ingredients.

## Figures and Tables

**Table 1:** Fitting results of Herschel–Bulkley equation for whey protein–citrate starch–capsaicin emulsion.

Temperatures	Whey Protein:Citrate Mung Bean Starch	τ_0_ (Pa)	K (Pa·s^−1^)	*n*	R^2^
25 °C	10:0	0.0137 ± 0.0002 ^a^	0.0147 ± 0.0002 ^d^	0.8534 ± 0.0026 ^e^	0.9990
	9:1	−0.0013 ± 0.0005 ^d^	0.0155 ± 0.0001 ^c^	0.9107 ± 0.0015 ^d^	0.9997
	7:3	−0.0006 ± 0.0001 ^d^	0.0063 ± 0.0000 ^f^	0.9911 ± 0.0005 ^c^	0.9993
	5:5	0.0079 ± 0.0002 ^b^	0.0156 ± 0.0000 ^c^	0.8455 ± 0.0007 ^f^	0.9992
	3:7	0.0042 ± 0.0005 ^c^	0.0102 ± 0.0001 ^e^	1.0321 ± 0.0012 ^a,b^	0.9997
	1:9	−0.0027 ± 0.0009 ^d^	0.0171 ± 0.0001 ^b^	1.0284 ± 0.0018 ^b^	0.9984
	0:10	−0.0009 ± 0.0010 ^d^	0.0221 ± 0.0001 ^a^	1.0361 ± 0.0011 ^a^	0.9989
50 °C	10:0	0.0052 ± 0.0000 ^a,b^	0.0046 ± 0.0001 ^c^	0.8633 ± 0.0029 ^e^	0.9971
	9:1	0.0068 ± 0.0002 ^a^	0.0027 ± 0.0000 ^e^	1.0367 ± 0.0040 ^b^	0.9978
	7:3	0.0036 ± 0.0002 ^b^	0.0036 ± 0.0000 ^d^	0.9055 ± 0.0009 ^d^	0.9960
	5:5	0.0045 ± 0.0001 ^b^	0.0036 ± 0.0000 ^d^	1.0074 ± 0.0009 ^c^	0.9987
	3:7	0.0051 ± 0.0004 ^a,b^	0.0044 ± 0.0001 ^c^	1.0047 ± 0.0030 ^c^	0.9981
	1:9	−0.0002 ± 0.0003 ^c^	0.0105 ± 0.0001 ^a^	1.0069 ± 0.0024 ^c^	0.9984
	0:10	0.0010 ± 0.0010 ^c^	0.0097 ± 0.0001 ^b^	1.0637 ± 0.0027 ^a^	0.9987

^a^ τ_o_ represents the yield stress, Pa; K represents the consistency coefficient, Pa·s^n^; n represents the fluid index; R^2^ represents the degree of fit. ^b^ All values are presented as mean ± standard deviation, and all of the measurements were performed in triplicate. ^c^ Different letters within each column at the same temperature indicate significant difference, *p* ≤ 0.05.

**Table 2:** The yield, encapsulation efficiency (EE), water, solubility, wetness, color, mean particle size, and size distribution of whey protein–citrate starch–capsaicin microcapsules.

W/S	Yield (%)	EE(%)	Moisture (%)	Solubility (%)	Wetness (s)	L*	a*	b*	ΔE	d _(4,3)_ (μm)	d _(3,2)_ (μm)	Particle Size Distributions (μm)		
												D_(10)_	D_(50)_	D_(90)_
10:0	74.99 ± 0.76 ^a^	94.18 ± 0.60 ^a^	1.38 ± 0.30 ^b^	96.32 ± 0.52 ^a^	162.72 ± 4.00 ^b^	93.38 ± 0.15 ^a^	2.86 ± 0.11 ^e^	16.60 ± 0.13 ^e^	0.78 ± 0.16 ^e^	1.12 ± 0.02 ^d^	0.83 ± 0.02 ^d^	0.46 ± 0.02 ^d^	0.96 ± 0.01 ^d^	1.99 ± 0.03 ^d^
9:1	58.98 ± 0.94 ^b^	91.51 ± 0.54 ^a^	1.59 ± 0.23 ^b^	87.97 ± 0.38 ^b^	174.18 ± 3.45 ^b^	93.49 ± 0.10 ^a^	2.75 ± 0.05 ^e^	17.03 ± 0.04 ^e^	0.87 ± 0.07 ^e^	1.20 ± 0.04 ^d^	0.87 ± 0.03 ^d^	0.47 ± 0.02 ^d^	1.08 ± 0.06 ^d^	2.09 ± 0.05 ^d^
7:3	57.54 ± 1.50 ^b^	90.21 ± 1.49 ^a^	2.40 ± 0.20 ^b^	80.69 ± 0.00 ^c^	172.61 ± 4.03 ^b^	92.59 ± 0.13 ^a^	3.78 ± 0.12 ^d^	19.56 ± 0.20 ^d^	3.70 ± 0.26 ^d^	32.30 ± 0.36 ^b^	5.36 ± 0.23 ^c^	5.17 ± 0.45 ^c^	33.47 ± 0.23 ^a^	57.13 ± 1.54 ^b^
5:5	48.06 ± 1.33 ^c^	81.53 ± 1.93 ^b^	1.65 ± 0.11 ^b^	72.74 ± 0.17 ^d^	211.73 ± 3.03 ^a^	90.48 ± 0.28 ^b^	7.77 ± 0.24 ^c^	22.63 ± 0.44 ^c^	8.95 ± 0.55 ^c^	32.60 ± 0.10 ^b^	6.45 ± 0.12 ^b^	5.87 ± 0.06 ^b^	32.87 ± 0.12 ^b^	58.80 ± 0.44 ^a,b^
3:7	31.00 ± 1.27 ^d^	58.65 ± 1.87 ^c^	2.03 ± 0.15 ^b^	68.88 ± 0.54 ^e^	223.59 ± 2.50 ^a^	86.98 ± 0.36 ^c^	11.15 ± 0.21 ^b^	30.26 ± 0.56 ^b^	17.81 ± 0.66 ^b^	27.70 ± 0.14 ^c^	6.61 ± 0.25 ^b^	5.17 ± 0.08 ^c^	28.45 ± 0.07 ^c^	48.75 ± 0.21 ^c^
1:9	19.63 ± 0.44 ^e^	26.59 ± 1.27 ^d^	3.63 ± 0.53 ^a^	65.97 ± 1.22 ^f^	218.45 ± 1.61 ^a^	83.55 ± 0.65 ^d^	14.87 ± 0.36 ^a^	34.86 ± 0.63 ^a^	24.58 ± 0.91 ^a^	33.37 ± 0.15 ^a^	7.55 ± 0.11 ^a^	6.45 ± 0.13 ^a^	33.40 ± 0.17 ^a^	59.97 ± 0.46 ^a^

^a^ W/S represents the ratio of whey protein to citrate mung bean starch. ^b^ EE represents the encapsulation efficiency. L*: lightness; a*: redness; b*: yellowness; Δ*E*: color difference. ^c^ All values are presented as mean ± standard deviation, and all of the measurements were performed in triplicate. Different letters within each column indicate significant differences, *p* ≤ 0.05.

## Data Availability

The data presented in this study are available on request from the corresponding author.
